# ‘Am I doing this right?’ Physician perceptions of the global assessment in clinical trials of systemic sclerosis

**DOI:** 10.1093/rheumatology/keaf377

**Published:** 2025-07-11

**Authors:** Hana Sabanovic, John D Pauling, Murray Baron, Laurence Clemens, Francesco Del Galdo, Christopher P Denton, Oliver Distler, Tracy Frech, Anna-Maria Hoffmann-Vold, Marie Hudson, Dinesh Khanna, Nancy Maltez, Thomas A Medsger, Peter A Merkel, Mandana Nikpour, Janet Pope, Virginia D Steen, Wendy Stevens, Elizabeth R Volkmann, Laura Ross

**Affiliations:** Centre for Health Policy, School of Population and Global Health, University of Melbourne, Parkville, Australia; Department of Rheumatology, North Bristol NHS Trust, Bristol, UK; Musculoskeletal Research Unit, School of Translational Health Sciences, University of Bristol, Bristol, UK; Division of Rheumatology, Jewish General Hospital, McGill University, Montreal, Canada; Department of Rheumatology, St Vincent’s Hospital Melbourne, Fitzroy, Australia; Leeds Institute of Rheumatic and Musculoskeletal Medicine, LIRMM, Leeds, UK; NIHR Leeds Biomedical Research Centre, Leeds Teaching Hospitals Trust, Leeds, UK; Centre for Rheumatology and Connective Tissue Diseases, Royal Free Hospital, London, UK; Division of Medicine, University College London, London, UK; Department of Rheumatology, University Hospital Zurich, University of Zurich, Zurich, Switzerland; Department of Medicine, Division of Rheumatology and Immunology, Vanderbilt University Medical Center, Tennessee Valley Healthcare System for Veterans Affairs, Nashville, USA; Department of Rheumatology, University Hospital Zurich, University of Zurich, Zurich, Switzerland; Department of Rheumatology, Oslo University Hospital, Oslo, Norway; Division of Rheumatology, Jewish General Hospital, McGill University, Montreal, Canada; University of Michigan Scleroderma Program, Division of Rheumatology, Department of Internal Medicine, University of Michigan, Ann Arbor, USA; Department of Medicine, Division of Rheumatology, The Ottawa Hospital, University of Ottawa, Ottawa, ON, Canada; University of Pittsburgh School of Medicine, Pittsburgh, PA, USA; Division of Rheumatology, Department of Medicine, University of Pennsylvania, Philadelphia, USA; Division of Epidemiology, Department of Biostatistics, Epidemiology, and Informatics, University of Pennsylvania, Philadelphia, USA; Sydney Musculoskeletal Research Flagship Centre, Faculty of Medicine and Health, University of Sydney, Sydney, Australia; University of Sydney School of Public Health, Sydney, Australia; Department of Rheumatology, Royal Prince Alfred Hospital, Sydney, Australia; Schulich School of Medicine and Dentistry, University of Western Ontario, London, ON, Canada; Division of Rheumatology, Department of Medicine, Georgetown University Medical Center, Washington, DC, USA; Department of Rheumatology, St Vincent’s Hospital Melbourne, Fitzroy, Australia; Department of Medicine, The University of Melbourne, Parkville, Australia; Division of Rheumatology, Department of Medicine, University of California, Los Angeles, Los Angeles, USA; Department of Rheumatology, St Vincent’s Hospital Melbourne, Fitzroy, Australia; Department of Medicine, The University of Melbourne, Parkville, Australia

**Keywords:** systemic sclerosis, outcome measures, global assessment, clinical trials, physician assessment

## Abstract

**Objectives:**

Physician global assessments (PhyGAs) are commonly performed in randomized controlled trials (RCTs) in SSc. However, there is no single PhyGA applied across RCTs. We performed an exploratory qualitative study to explore perceptions of the PhyGA, its role in RCTs and how physicians perform their own assessment.

**Methods:**

Participants with expertise in the clinical assessment and, or actively involved in research on SSc were invited to participate. Participants were asked to define disease constructs of activity, damage, severity, and overall health, and to describe how they perform a PhyGA and their perception of what a PhyGA should assess. Interview transcripts were analysed using deductive and inductive thematic analysis.

**Results:**

Eighteen rheumatologists and one patient research partner were interviewed. Four major themes were identified: (i) physician uncertainty; (ii) variation in the conduct of a PhyGA; (iii) physician efforts to improve PhyGA consistency; (iv) utility of a PhyGA. Most participants felt a PhyGA should assess changeable aspects of SSc, commonly conceived of as disease activity. There was considerable uncertainty about the optimal method for assessing disease activity. Participants were uncertain about their own methods of performing a PhyGA, and variability in the application of the instrument was identified. Despite these limitations, physicians generally agreed that the PhyGA is useful and can assess unquantifiable aspects of SSc.

**Conclusion:**

We identified significant heterogeneity in the approach to PhyGAs in SSc. This variation was considered a limitation of the PhyGA. Overall, a PhyGA was viewed as a useful instrument that can aid the assessment of treatment response in RCTs.

Rheumatology key messages

- There is no single physician global assessment (PhyGA) applied in clinical trials of systemic sclerosis.
- Physicians believe a global assessment is a useful tool for measuring overall patient status.
- Variability in the application of the PhyGA is a limitation of the instrument.

## Introduction

SSc is a multi-system autoimmune disease associated with high morbidity and mortality, for which there are few effective treatments [1]. The clinical heterogeneity of SSc has made it challenging to identify disease-specific treatment targets and design randomized controlled trials (RCTs). The lack of valid, global SSc outcome measures may have contributed to the failure of recent RCTs to prove the efficacy of novel treatments [2, 3].

Across many rheumatic diseases, global assessments are widely used to assess treatment response [4, 5]. Global assessments refer to single-question instruments used to measure the disease in its totality across all affected organ systems. Both patient and physician global assessments are widely used in RCTs, either alone or as part of composite response indices [6]. However, there is no standardized, universally applied physician global assessment (PhyGA) used in SSc RCTs [7]. For example, recent RCTs have variably asked physicians to rate one of the following disease constructs: current disease activity; disease severity/overall impact of disease over past 7 days; a global assessment of disease; or assess current SSc status. No single disease construct is consistently assessed by the PhyGA. Concerns have been raised about the reliability of the PhyGA given the lack of standardization of both wording and item responses [5, 7–9]. Assessment of the concurrent use of three PhyGA instruments, each worded to assess activity, damage, or overall health, in a cohort study showed that the results of each PhyGA were not directly interchangeable and that each PhyGA was influenced by the presence of different SSc features and comorbidities [10]. These findings highlighted the need to develop a standardized PhyGA to apply consistently across all SSc RCTs.

The Scleroderma Clinical Trials Consortium (SCTC) has convened a working group to develop a SSc-specific PhyGA that can be used to assess response to therapy and discriminate between treatment arms of an RCT. Following initial working group meetings, it was apparent there were several views of the PhyGA and divergence in opinion regarding the disease construct that should be assessed with a PhyGA. Therefore, we undertook this study as the first step towards developing a standardized PhyGA for application in RCTs.

## Methods

### Study design

A qualitative design underpinned by phenomenology and using semi-structured interviews was chosen to better understand individual physician perceptions of the PhyGA in clinical trials and how they perform their PhyGAs. This study is reported in line with the Consolidated Criteria for Reporting Qualitative Research (Supplementary Data S1) [11].

### Participant recruitment

SCTC members were invited to participate in the SCTC Global Assessments Working Group and form a project steering committee. The project steering committee included 11 rheumatologists and two patient – research partners. Working group members were selected based on their experience and expertise in outcome measure design and implementation of SSc RCTs [12].

All project steering committee members, including patient – research partners, were invited to participate in an online interview by personal email in July 2023. The patient – research partners were invited to participate to obtain their perspective on physician assessments in clinical trials. One physician and one patient – research partner declined to participate. A purposive sampling strategy was then applied to recruit additional participants to ensure representation of rheumatologists (referred to as physicians in this report) across different career stages and from varied geographic locations between September and November 2023. Additional participants were selected based on their recognized expertise in the assessment of SSc. All additional invited participants agreed to be interviewed. Recruitment ceased when thematic saturation was reached, meaning no new themes were identified from analysis of the interview transcripts [13].

### Development of interview guide

Four non-steering committee member rheumatologists participated in a structured group discussion focused on global assessments, what they measure and how they are performed. The results of this discussion were used to develop the study interview guide (Supplementary Data S2). The results of this interview were not included in the analysis.

Topics covered in the study interviews included (i) the definition of disease activity, severity, damage and overall health; (ii) the aim of the PhyGA and which disease construct it should measure; (iii) how a PhyGA is performed; (iv) the perceived shortcomings of the PhyGA. Participants were asked to consider the role of a PhyGA in a clinical trial, with an explicit focus on the use of a global assessment in this context, rather than routine clinical practice. Topics covered in the patient – research partner interview were modified. Topics covered included: (i) definition of disease constructs; (ii) perceived aims of a PhyGA; (iii) perceived shortcomings of physician assessments in a clinical trial.

A disease construct was considered an aspect, or concept, of disease that can be measured in an RCT. The disease constructs of activity, severity, damage, and overall health were included in this project, because they represent the global disease concepts frequently measured by PhyGAs in SSc RCTs [7].

### Interview procedure

All interviews were performed by one female study investigator [L.R. (MBBS, PhD] who specializes in SSc and is a member of the SCTC, with the assistance of an experienced female qualitative researcher [H.S. (MIP, MPH, Research Assistant)], who has an interest in chronic disease. Assistance provided included design of the overall study procedures, development of the interview guide, interview procedures and data analysis, and presentation. Single, paired or small group (*n* = 3) interviews were conducted between September and December 2023. Each participant was interviewed once. No more than three participants were interviewed at a single time to allow each participant adequate opportunity to express their opinions within a feasible time frame. All participants were provided with written information at the time of invitation about the purpose of the interview. At the commencement of the interview, participants were verbally re-informed of the overall project aims, the specific purpose of the interview, and L.R.’s background and research interests. Participants were invited to make additional comments during each interview to ensure all topics they wished to discuss were covered. Academic rigour was maintained through note-taking following each interview and reflexive practice meetings. All participants verbally consented to participate in an online video- and audio-recorded interview (Zoom teleconferencing platform) and to the use of de-identified data in published reports.

### Data analysis

Interview recordings were directly transcribed verbatim by the Whisper application (OpenAI) and checked by a study investigator (L.R.) against the audio and video interview recording and interviewer notes. Transcripts were imported into QSR NVivo 12 for coding. Data analysis was conducted using a combination of inductive and deductive thematic analysis [14, 15]. Two study investigators (H.S., L.R.) developed a coding tree based on the structure of the interview guide (deductive analysis) and analysed five interview transcripts independently. Through this process, more codes were identified, and these were compared, discussed and refined through discussion (inductive analysis). A single study investigator (H.S.) coded the remaining interview transcripts according to the agreed coding tree. Additional codes were added as they were identified. Similar codes were grouped together and collapsed into themes. Themes were discussed until consensus was reached. Data from all interview transcripts, including the interview with the patient – research partner, were included in the final analysis. Data and exemplary quotations that were identified in the patient – research partner interview are presented, together with the data from the physician interviews. Participants were provided a manuscript draft and all provided feedback.

## Results

Seven individual participant interviews, five paired participant interviews and one group interview of three participants were conducted. Interviews averaged 65 min (range 27–98 min) in length. Physician participants had a varied length of clinical practice; two (11%) had <10 years practice, 5 (45.5%) 11–20 years practice, 5 (45.5%) 21–30 years practice and 6 (33%) physicians >30 years practice. The demographic characteristics of the participants are outlined in Table 1. Four major themes were identified in the dataset (Table 2).

**Table 1.** Participant characteristics

Participant number	Gender	Geographic location	Interview method
1	Male	Europe	Group
2	Male	North America	Group
3	Female	Asia Pacific	Group
4	Female	North America	Paired
5	Male	North America	Paired
6	Male	UK/Europe	Individual
7	Female	North America	Individual
8	Female	UK/Europe	Paired
9	Male	UK/Europe	Paired
10	Male	North America	Paired
11	Female	North America	Paired
12	Male	UK/Europe	Individual
13	Female	North America	Individual
14	Male	Asia Pacific	Paired
15	Female	Asia Pacific	Paired
16	Female	North America	Individual
17	Male	North America	Individual
18	Male	Asia Pacific	Individual
19	Female	North America	Individual

**Table 2.** Summary of thematic analysis

Theme/Subtheme	Exemplary quotation
**Uncertainty about the PhyGA**	
1.1 Complexity of SSc contributes to physician uncertainty	A. ‘If someone goes into heart failure and their skin improves, or has pericarditis and cardiomyopathy, those are very different issues. And so how do you weigh the overall global?’ –Participant 11 B. ‘I try and weigh up, well, you’ve got really bad gut, incontinence and all that, some digital ulcers, but you haven’t got bad lungs but you feel really crap, and this is where I find it really hard, how do you weight that against someone who’s got all of those and pulmonary hypertension?’ – Participant 15
1.2 Uncertainty about differentiating between disease activity and damage	C. ‘If a patient has a lot of diarrhoea or a patient has a lot of faecal incontinence, I would most likely also say it’s high disease activity, despite knowing that maybe it’s just damage. So, it’s not that easy for each organ to know the difference between active disease and damage.’ – Participant 8 D. ‘You can also have damage in one organ but still-active disease in another organ, and you can have still-active disease and damage in the same organ.’ – Participant 8
2) **Variation in the conduct of a PhyGA**	
2.1 Variable opinions about construct measured by PhyGA	E. ‘I am probably looking at activity and some influence of severity without being really clear how much influence each of those components [activity and damage] has.’ – Participant 1 F. ‘I just automatically think of it as severity, you know, is there activity going on? But also, have they accrued so much damage that they could have end-stage lung disease, they’re on home oxygen. But, you know, the disease is quiet, hasn’t changed in 5 years.’ – Participant 2 G. ‘I think most of the clinicians and patients are looking at that global, but interpreting it as an activity scale, more than a damage scale. Otherwise those things wouldn’t change.’ – Participant 5 H. ‘I don’t think there is a particular disease construct [measured by a global]’ – Participant 6 I. ‘I just answer, I don’t care what the question says, I’m answering about activity because otherwise it won’t change.’ – Participant 4
2.2 Inconsistent opinions about inclusion of subjective impressions	J. ‘I think it does [include subjective impressions] in the personal choice of that physician. And so, if I decide to escalate treatment or not, that is an element. But I’m wary of giving that a weight for an FDA (U.S. Food and Drug Administration) approval’. – Participant 12 K. ‘I would look beyond just like the interstitial lung disease but look for other possible contributing factors. So this could be other disease manifestations like, say, pulmonary hypertension. It could be other factors related to their lifestyle, like deconditioning. I really look at the whole patient. So not just even the scleroderma but what’s going on in their home life, with their job, all these other factors that can influence how they feel … but I would look beyond that narrow scope of the fibrosis in the lungs.’ – Participant 20
2.3 Disagreement about inclusion of patient-reported symptoms	L. ‘If the patient has a lot of pain, fatigue, sleep, disability, even if they have mild organ involvement that would influence where I put on a zero to 10 scale.’ – Participant 5 M. ‘I think my global … I mean a lot of things are going through my mind in that moment, you know, including the patient, how they’re functioning and how it’s affecting their life and also their response to treatment and what treatments that they’ve required and how they’ve responded to that, and then that in combination with what I see on physical exam and testing.’ – Participant 13 N. ‘I’m not sure that I’m always taking [symptoms] into account … if I have to do a physician global … I really think about the organs affected by scleroderma. And fatigue to me is not an organ. So I wouldn’t integrate fatigue, even though this patient may be tremendously [affected], that wouldn’t fit into my impression of the global assessment. Same thing with pain. Pain is not something that I can palpate, so I’m not sure that I would include pain.’ – Participant 16
2.4 Differing reference points for the physician global assessment	O. ‘I’m just going relative to the worst cases I’ve ever seen on activity and relative to that it’s lower.’ – Participant 4 P. ‘There’s an interesting concept of the case mix. So someone like me, where 80% of the patients that I see will have SSc, that would be a little bit different from perhaps a much more frequent situation of a general rheumatologist, where 90% of the patients they saw would not have SSc, but they might have lupus or vasculitis or FM or other conditions … that could be very relevant because I would be putting my physician global very much in the framework of SSc patients. Whereas someone else might broaden it a bit and be thinking about physician global in a broader population of disease.’ (Participant 6) Q. ‘I think that the best control is the way that patient was maybe before they got their disease or very early on in their disease and then each 3 to 6 months where are they progressing to? Because, as we’ve said, I mean one person’s bad scleroderma might be another person’s ‘oh I can manage that’. It depends on what you’re comparing it to for that particular patient, and I always think that, I always look at what they are expecting and what they were able to do in the past and what they’re hoping to be able to do in the future.’ – Participant 14 R. ‘I think it depends on how long they are into their disease, right? If it’s within the first few years, yes, but then beyond that point, like it seems like we create new baselines, right?’ – Participant 20
3) **Physicians’ efforts to improve consistency of PhyGA**	
	S. ‘I’m walking through the organs. I’m walking through inflammation on labs. I really walk through the patient in my head, what kind of organs are involved, how heavily involved, do they have inflammation?’ – Participant 8
4) **Utility, and limitations, of a PhyGA**	
	T. ‘A physician global assessment in scleroderma means nothing because it’s a physician global assessment of what? Of activity? Of severity? Of damage? Of overall health? And you know it goes on …’ – Participant 16
	U. ‘I think it’s useful as an anchor … Well, there’s several functions, but one is as a sense check, you know, to kind of make sure that you’re not in a multi-compartment heterogeneous disease going to be completely kind of misled in a sense by spurious assessments of one dimension of disease.’ – Participant 6

PhyGA: physician global assessment.

### Theme 1. Physician uncertainty about the physician global assessment

#### 1.1 Complexity of SSc contributes to physician uncertainty

SSc was described as a complex, multifaceted disease that is incompletely understood. Physicians were challenged by the frequent discordance in presentation and rate of change across various organ systems. This made it difficult to give an overall global score. (Quote A, Table 2) Nearly all physicians expressed uncertainty about how they perform a PhyGA. Gaps in scientific knowledge about SSc and a lack of standardization of the PhyGA contributed to uncertainty.

Physicians found it difficult to determine what was meant by active disease (Quote C, Table 2). Without clear biomarkers for activity, particularly of fibrotic and vasculopathic disease manifestations, physicians were looking for the ‘outward manifestations of that process’ (Participant 2). In the absence of overt disease manifestations, such as digital ulcers or renal failure, physicians could not determine with certainty whether a patient had active SSc. Furthermore, physicians commented on the variability in presentation of states of high disease activity between patients and between various organ systems (Quote B, Table 2). Most participants expressed self-doubt in their ability to perform a PhyGA, frequently asking, ‘Am I doing it right?’ (Participant 7). Only a small minority of physicians conveyed confidence in their approach.

#### 1.2 Uncertainty about differentiating between disease activity and damage

Physicians discussed how both activity and damage can be simultaneously present, and how current tests cannot distinguish between them (Quote D, Table 2). Disease activity was conceptualized as aspects of SSc that are changing and potentially responsive to treatment. Physicians looked for biomarkers of inflammation, disease progression, and results of therapeutic trial and error to identify disease activity. Damage was conceived of as irreversible changes that commonly result in impaired organ function. Damage was considered a result of disease activity, and most participants considered activity a construct distinct from disease damage (see Supplementary Data S3 for examples of physician definitions of activity and damage).

### Theme 2. Variation in the conduct of a physician global assessment

#### 2.1 Variable opinions about construct measured by physician global assessment

There was significant variability in the interpretation of a PhyGA question and the implementation of the instrument. Unless specifically instructed, most physicians thought they were being asked to rate disease activity in an RCT, because activity predominates in early disease, can be modified by effective therapies, and because severity or damage ‘is not sensitive to change in a trial*’* (Participant 4) (Quote G, Table 2). Others considered the construct of severity—conceptualized as a combination of activity and damage—when performing a PhyGA, and some considered all of activity, damage and severity (Quote E and F, Table 2, see also Supplementary Data S2 for definitions of activity, damage and severity). A minority of physicians did not have a specific construct in mind when making their assessments (Quote H, Table 2).

There was a general perception that other physicians were scoring activity when performing a PhyGA (Quote G, Table 2). Some physicians stated they rated disease activity even if the question posed in the RCT had a different focus (Quote I, Table 2). However, they were a minority, and most were guided by the wording of the PhyGA question. Participants agreed that if a global assessment asked about a patient’s overall health, this assessment would include consideration of SSc and any comorbidities and treatment complications. Though ‘there’s massive overlap’, SSc status and overall health were considered ‘separate’ (Participant 18) by both the physicians and patient – research partner. Overall health was considered an inappropriate construct to measure in an RCT, owing to the potential effects of comorbidities on a patient’s presentation (see Supplementary Data S3 for definitions of overall health).

#### 2.2 Inconsistent opinions about inclusion of subjective impressions

Many physicians described including their subjective impression of a patient when making clinical assessments. Subjective impressions were commonly described as ‘a feeling of what the status of the patient is at any particular time’ (Participant 10) and a skill that matures with experience. Some physicians felt that subjective impressions were an inherent aspect of a PhyGA. However, others did not think it appropriate to incorporate subjective impressions in their assessment, especially in the context of an RCT. They indicated that, ‘You’re always a little bit more rigorous in your thought processing’ (Participant 6) in this setting, because an FDA approval should only be reliant on data that can be quantified ‘like physical exam findings and labs and imaging’ (Participant 7). There were no notable differences between physicians who knowingly included their subjective impressions in their PhyGA and those who did not, but physicians who did were more likely to take patient-reported symptoms into account when performing a PhyGA.

#### 2.3 Disagreement about inclusion of patient-reported symptoms

Most physicians took psychosocial factors—such as impact of disease on work participation, effect of disease on mood, and dependence on caregivers for daily assistance—into account when making an assessment. Some physicians also considered patients’ report of symptoms such as fatigue and pain, but generally only when daily living was impacted (Quotes L and M, Table 2). Physicians felt patients were more likely to be concerned about daily function and how they feel, while physicians were more likely to focus on disease manifestations linked to mortality. Many physicians described ‘taking into account what is influencing the overall outcome [mortality] of patients … which maybe explains why the physician globals are so different to the patient globals, because they rate these things very differently’ (Participant 1).

Physicians noted that patients’ self-assessments were subjective because ‘everybody’s impact of disease is different’ (Participant 2), and patients’ impression of impact could depend on how long they had been living with SSc and their personality. These physicians did not include an assessment of patients’ symptoms in their PhyGA, because the patient global assessment ‘get[s] all of the subjective stuff’ (Participant 7).

#### 2.4 Differing reference points for the physician global assessment

Most participants judged the patient they were assessing by reference to the total SSc patient population; in other words, they compared their current patient to ‘that very worst patient who is bed-bound and about to die, on oxygen and has a bad heart’ (Participant 16). Physicians reported that the boundaries of this population changed ‘with experience [as they saw] more patients with SSc’ (Participant 13) and on the type of rheumatologist who was doing the assessment (i.e. generalist *vs* SSc specialist) (Quote P, Table 2). Some participants considered both the stage of disease and specific disease manifestations when performing their PhyGA (Quote R, Table 2). Other participants found it problematic to compare a patient’s status with that of other patients owing to the significant variability of disease manifestations, daily impact and patient adaptation to the disease. These participants preferred to assess a patient compared with how they were at the last visit or, if early on in their disease, to how they were prior to being diagnosed with SSc, describing *‘*It’s easier actually [comparing patient’s condition with that on the previous visit], and it makes a bit more sense than trying to give a global that is crossing all these boundaries of different severities and different organs and different tissues. What you really want to know is whether they are improving as a result, or worsening as a result, of your intervention’ (Participant 14). The choice of reference point used for comparison appeared related to the physicians’ conception of whether the purpose of the PhyGA is an absolute measure of disease status or whether it is a relative measure of change over time.

### Theme 3. Physician efforts to improve the consistency of their global assessments

All physicians noted they had not been trained how to perform a PhyGA and described themselves as self-taught. In the absence of a standardized PhyGA, most physicians described an internal protocol ‘that has changed over the years’ (Participant 8) to ensure their own consistency between patients. This enabled them to know ‘what that [score] means for me’ (Participant 13), even if it is not standardized against that of other physicians. These individual protocols were similar among physicians and generally consisted of a checklist of all affected organ systems, and consideration of the severity of organ involvement and the rate of change of each disease manifestation (Quote S, Table 2). Some physicians limited their checklist to the top three organs affected.

A number of physicians described how this internal protocol changed depending on whether they were reviewing a patient in the early or later stages of disease, with the musculoskeletal system and skin prioritized in the early phase of illness and a greater weight given to cardiopulmonary manifestations as the disease progresses. ‘The musculoskeletal and skin predominates my global early on, whereas later on, 5 years down the road, the skin has improved … but the forced vital capacity is declining’ (Participant 5). While the PhyGA was influenced by the prognostic importance of disease manifestations for most physicians, describing ‘those manifestations associated with worse prognosis, I do tend to weight more’ (Participant 13). A minority did not include the prognostic importance of organ involvement in their assessment, because they felt that it was too difficult to predict how an individual patient will progress. For others the prognostic importance of a disease manifestation mattered only over a certain irreversible threshold, such as ‘a patient who has pulmonary arterial hypertension, functional class four, is on triple therapy, and has very poor function and perhaps a prognosis of 2- or 3-years survival; that patient is always going to have a very high physician global from my point of view’ (Participant 6). Physicians preferred to have the latest investigation results available when making a global assessment.

### Theme 4. Utility, and limitations, of a physician global assessment

Physicians tended to agree that the PhyGA is a valuable tool that provides a holistic perspective of a patient’s wellbeing that captures something greater than the ‘individual components … such as lung, skin, gastrointestinal tract, in ways that we have not yet mastered’ (Participant 20). The PhyGA was widely considered to be useful for comparing the clinical status of individual patients with the whole spectrum of SSc, because it could provide insight into a patient’s disease trajectory and act as a ‘commonsense check of other investigations’ (Participant 6). Universally, participants identified that the SSc PhyGA needs to be standardized, and many researchers indicated training in how to perform a global assessment might be helpful (Supplementary Data S4). Physicians highlighted that the lack of standardization of the instrument meant the PhyGA was fallible to inter-rater variability, with a perception that each physician performs their global assessment differently and ‘imagining 10 physicians independently judging the same patient and coming up with inherently different judgments’ (Participant 6).

Participants felt the PhyGA could function as a measure of activity, used in the absence of validated biomarkers; ‘we don’t have great direct measures of what that process is, so we rely on all these secondary measures that we observe or are told by the patient’ (Participant 1). However, all physicians were aware of and acknowledged the limitations of the PhyGA discussed above. For a minority, these limitations raised doubts as to the PhyGA’s overall usefulness. Some physicians questioned the use of a single instrument for a heterogeneous multi-organ disease such as SSc. Instead, they suggested it may be more appropriate to use instruments relevant to the specific intervention or disease manifestation being studied, to provide ‘more granular measures by organ system’ (Participant 16).

## Discussion

This is the first study to present physicians’ perceptions of PhyGAs in SSc. The results of this study will be used in the PhyGA standardization process. In the absence of reliable measures of overall disease status in SSc, most physicians considered a PhyGA a useful tool for supplementing examination and investigation findings A PhyGA was reported to be a useful commonsense check of other outcomes. Most physicians thought a PhyGA should focus on the assessment of disease activity, because measurement of activity is what will lead to the identification of new, effective treatments for SSc. However, global assessment of SSc is associated with significant clinical uncertainty, and there is widespread variation in the interpretation and implementation of PhyGAs.

Physician uncertainty and concern about the individual ‘correctness’ of their own assessment arose in all interviews and perpetuated the widely held view of the high variability associated with PhyGAs. Most physicians had independently adopted a decision-making approach to manage the clinical uncertainty inherent to making a PhyGA [16]. Physicians applied such strategies as a way of managing assessments of the unknown or unmeasurable aspects of disease and to ensure a degree of rigour and internal reproducibility in their PhyGAs. The application of judgement to aid the interpretation of an individual’s presentation in the context of all a physician understands is characteristic of the experienced physician [17–19]. These judgements can offer insights that are unable to be measured by examination or investigation findings alone; however, they are associated with an inherent risk of over-reliance on heuristics and biases [20]. Physicians frequently regard clinical interpretation as second class to objective evidence of disease status [17]. Physicians’ reflections about the potential limitations of the PhyGA highlighted a core tension in clinical medicine—that physicians are always working in situations of uncertainty despite being trained to aim for maximal certainty [17, 19]. This can be considered both a strength and a weakness of any PhyGA.

**Figure 1.**
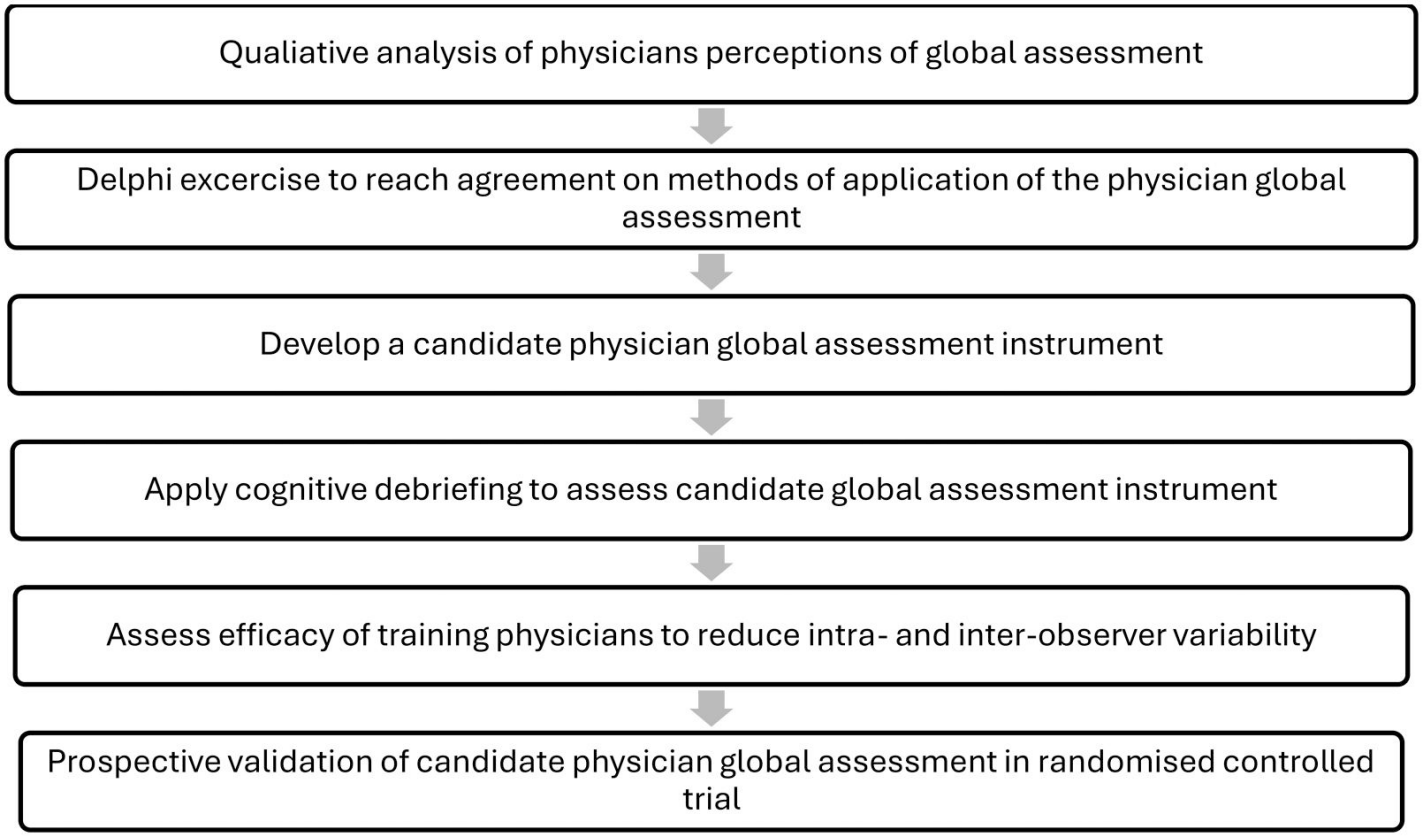
Proposed methods for standardization of a SSc physician global assessment instrument

Standardization of the PhyGA was viewed as a vital step towards resolving much of the uncertainty and variability associated with the instrument. Our results indicate there needs to be standardization of item wording, response options and recall period, and of how the PhyGA should be performed in an RCT, with agreement on what contextual information is made available at the time of scoring. Heterogeneous application of global assessments is a recognized problem across many rheumatic diseases, with the psychometric properties of PhyGAs in the field of rheumatology largely untested [5, 21, 22]. In the field of SLE, the lack of standardization of the PhyGA is recognized [5]. Current efforts are underway to achieve consensus as to how the PhyGA can be applied in SLE RCTs in order to improve the reliability of the instrument’s results [23]. Standardization of the PhyGA in other rheumatic disease has not yet occurred. To address this issue of standardization of the PhyGA in SSc, the next stage of this project is to survey the opinion of global SSc clinicians and researchers via the SCTC and European Scleroderma Trials and Research Group networks to reach agreement about the construct measured by a PhyGA and its method of application in RCTs (Fig. 1).

Participants raised the potential need for training to improve the reliability of the PhyGA. There is no precedent for the training of physicians in making a global assessment. Training can improve the reliability of outcome measures such as the modified Rodnan Skin Score [24, 25]. It was hypothesized by many participants that PhyGA training prior to the commencement of any clinical trial, with explicit instruction on what aspects of disease to consider, may make the PhyGA a more rigorous outcome measure. The effect, if any, of training on physician global responses will be assessed in future stages of this project.

Strengths of this study include the systematic evaluation of the perspectives of a diverse group of participants from a range of geographic locations and at various career stages. Thematic analysis has identified key issues pertaining to the standardization and validation of the PhyGA that can be addressed in future steps. However, this study is not without limitations. We did not observe differences in responses according to geographic location or gender. This may be because most of the participants interviewed had >10 years of practice and came from English-speaking countries. All participants noted that the way they conduct their global assessment was refined over time. Future studies should compare the PhyGAs of early-career rheumatologists with those of more experienced rheumatologists, and evaluate whether cultural background and/or location of practice influences the PhyGA. Only one patient was interviewed in this study. This was intentional, given the PhyGA is a physician-reported outcome. No additional themes relevant to this paper arose from this study, and no further patients were interviewed. It is acknowledged that there may be divergent views among the SSc patient population of how physicians should assess SSc, and more themes may have arisen if a larger group of patients had been interviewed. A single SSc patient interview does not allow for any analysis of physician responses in relation to patient experiences of SSc. To fully ascertain patient perspectives of global assessments in SSc clinical trials, a further study that elicits a range of patient perspectives across varied geographic locations and experiences of health systems and disease manifestations is required.

The steering committee members are all individuals who have an interest in and expertise in the development of outcome measures and are involved in the design of SSc clinical trials. This places the results of the study at risk of bias, owing to the particular clinical experiences associated with an academic rheumatology practice and the participants’ professional interest in changes to SSc clinical trial design. This potential bias has been somewhat mitigated by the inclusion of external, non-committee member participants. Additionally, the interview guide was developed in consultation with a qualitative researcher, following an exploratory discussion with four non-committee member rheumatologists. Although committee members were aware of the overall aims of the project and had agreed on the need for further investigation of physician perceptions of the PhyGA, all interviewees were ‘blinded’ to the interview structure and questions at the time of their study participation.

In conclusion, there is no universal conceptual understanding, nor a universal application, of the PhyGA in SSc. However, physicians believe a global assessment to be a useful outcome measure of overall disease status, and should be used to assess disease activity in the context of an RCT. Even though there are instruments for measuring each of activity, damage and severity in SSc [26–29], the PhyGA was thought to add information not captured by other assessment techniques. Physicians consistently express a desire for a standardized approach to the PhyGA to apply in RCTs, to improve the quality and robustness of study results. This is a mandatory step to ensure the validity of the PhyGA itself and that of composite outcome measures that include global assessments [6]. Work to standardize a SSc PhyGA, informed by the physician perspectives and experiences identified in this study, is currently underway.

 Ethics approval for this study was provided by the Human Research Ethics Committee at St Vincent’s Hospital Melbourne (LRR 040/23).

## Supplementary Material

keaf377_Supplementary_Data

## Data Availability

The data underlying this article will be shared on reasonable request to the corresponding author.
