# Supplementary material for: ‘Am I doing this right?’ Physician perceptions of the global assessment in clinical trials of systemic sclerosis
Source: Rheumatology (Oxford). 2025 Jul 11;64(11):5844–52. doi: 10.1093/rheumatology/keaf377 (PMC12596065; doi:10.1093/rheumatology/keaf377)
Supplement: keaf377_Supplementary_Data [file keaf377_supplementary_data.zip › keaf377_Supplementary_Data/rhe-25-0923-File004.pdf]

Supplementary Index 2: Physician Global Interview Guide

Introductions

Study outline

- State goals of the project
- State aim of this focus group meeting: To discuss the physician global assessment, as it is performed in a randomised controlled trial. I would like to understand what you think is the 'global assessment' of a scleroderma patient. And then consider what idea, or construct, you think we should be assessing when we ask clinicians to make a global assessment in a RCT.

Initial questions:

What does disease activity in scleroderma look like?

- When did you last see a patient with active scleroderma? What did they look like?
- How did you ascertain they had high disease activity?
  - What disease features did you look for?
  - What investigation results did you look at?
- Did you consider how they looked last time you saw them?

What does disease damage look like in scleroderma?

- What does a patient with severe damage look like?
- How do you determine that this patient has any damage?
  - How do you assess degrees of damage (i.e. low, medium or high level of damage)?
- Do you consider investigation results when making an assessment of disease damage?

Can disease activity and damage be present at the same time?

What does disease severity mean in scleroderma?

- What does a patient with highly disease severity look like?
- How do you assess disease severity?

When asked to complete a physician global assessment is there a particular disease construct that is most important to you?

- Do you think about activity or damage or severity?
- Do you think about test results?
- Do you think about patient's symptoms?
- Do you think about effect of scleroderma on patient's function?

When you assess overall disease, do you have a checklist of items you consider?

- Do you have an 'internal' / personal protocol you follow when assessing overall disease?
- Is there an order of disease features you assess?

- Is there a relative importance of disease features (e.g. are investigation findings more important than function? Or function more important than symptoms?)

Is there a relative weighting of items that you assign for various disease manifestations?

Is time frame of assessment period important?

Do you need to have investigations present / reviewed before you can make your global assessment?

- Should this be standardised?
- Is it possible to make a global assessment without investigation results?

How do you assess change in disease in scleroderma?

- What makes you change, either positively or negatively (improved / not improved), your global assessment?

What is your reference point when making a global assessment?

e.g. SSc patient population in general, health controls, reference to individual patient when they were in good health, patient at previous review?

Who taught you how to perform a physician global assessment?

- How did you learn to perform a global?
- Do you think your clinical experience affects the way you perform a global?

Should we have / Do we need a physician global assessment?

- What is the role of the physician global?
- What does it add to disease assessment?

Do you read the question given in a RCT?

Is the setting important? Does your global assessment change if you are making the assessing in a clinical trial vs observational study vs routine clinic visit?

Has your opinion of the physician global assessment instrument changed after discussion?

- What are reasons for change?

Supplementary Index 3: Physician definition of constructs used to make global assessments for systemic sclerosis

Disease construct and their descriptions	Exemplary quotation
**Disease activity**	
Reversible	'Reversible manifestations are the easiest to say, oh yes, this is active.' – Participant 13
Evident in early disease	'In early disease, I think I think that's how I look at activity in scleroderma has to be early immune inflammatory phase for me to call something as an active disease.' – Participant 5
Inflammatory	'For me, activity almost needs to have an inflammatory component, that there has to be active ongoing inflammation.' – Participant 16
Dynamic disease worsening	'So, it could be worsening the gastrointestinal tract, or worsening of the lung disease, or worsening of the skin disease, or worsening of the vascular disease.' – Participant 2
Driver of damage	'I think I'm using progression of damage as a construct that helps me to assess activity as long as I do not have cross-sectional markers telling me when the patient is sitting in front of me if the patient is active and will progress in the future. Because I think continuous activity has the danger of progression of damage.' – Participant 1
May not lead to damage	'I mean, we accept synovitis, I think, as being activity. You have it because they have inflammation going on at the joints, or around the joints, right? It doesn't always lead to damage, it can go away on its own, and then the activity has stopped, you know?' – Participant 2
Change over time	'I think I've come to the conclusion that you cannot assess disease activity just with a patient in front of you as a snapshot. So, for me it is delta that is important.' – Participant 9
Responsive to treatment	'…If you have effective disease modifying treatment, I would expect that the activity should definitely be the compartment of the disease you would very, very much expect to get better and maybe you know with an effective treatment get rid of completely.' – Participant 6

Disease construct and their descriptions	Exemplary quotation
Clinical features of disease activity	‘I’m also listening to the patient actually because when the patient is feeling worse… I also assume there’s activity.’ – Participant 1<br><br>‘Early disease with progressive skin, tendon friction rubs, activity inflammatory arthritis, myocarditis, early, early NSIP, ground glass predominant ILD.’ – Participant 5<br><br>‘A patient who was having a scleroderma renal crisis, I would say, well, you’d have to put that in the category of a patient with active disease…An acute vascular problem – so gangrene critical ischemia, a very severe digital ulceration, would also be a patient who I would consider to have very active disease.’ – Participant 6<br><br>“Tendon friction rubs an example, for me, it’s always disease activity…Inflammation on the labs with CRP or ESR or high platelets or whatever, then for me that’s clear activity.’ – Participant 8
**Disease damage**	
Irreversible	‘Even if you took the scleroderma away the organ has been irreversibly damaged so it is that concept of irreversible damage, consequence of the disease that might still persist even if you took away the pathology of the condition.’ – Participant 6<br><br>‘I conceive damage the part of scleroderma that cannot be reversed.’ – Participant 19
Anatomical deformities	‘…the most obvious form of it is people who do have physical deformities.’ – Participant 14
Unresponsive to therapy	‘With damage, the horse is already out of the barn, it's already happened. I'm not giving any therapies per se. I mean, those are the patients still you would do like occupational therapy, physiotherapy, pulmonary rehabilitation. You may have a decision-making plan, but you less are inclined to think that your immunomodulatory immunosuppressive therapies are going to work.’ – Participant 7
Loss of function	‘…loss of function really, I mean either physical function or organ function, I think that's really where I'm thinking about damage.’ – Participant 6

Disease construct and their descriptions	Exemplary quotation
Consequence of activity	'I definitely think it's different from activity. It's to a large extent the result of activity.' – Participant 2
Later stage of the disease	'I think all of us would agree that most of the latter part of scleroderma is damage. So, damage would look like claw hand deformities patients, where you can do whatever, you can soften the skin, but the claw hand deformities are left.' – Participant 5
Inherent aspect of SSc	'But irreversible damage as a baseline which all scleroderma patients have to a certain extent, even in early stages, you're dealing with fibrotic change, which is not fixable.' – Participant 10
Static	'…When I think about damage, it's something that's more static and activity is more dynamic.' – Participant 20
Clinical features of disease damage	'Pulmonary arterial hypertension…. I think of it as a lot of damage.' – Participant 4
	'UIP or a lot of fibrotic ILD that whatever you do is not going to reverse the damage. Gastrointestinal (symptoms) always a severity and damage…Those calcinosis are not reversible.' – Participant 5
	'Those patients with very severe deformed hands, wrists, very poor hand function, very poor loss of muscle, bulk and muscle strength, not due to inflammation, but really as a consequence of scarring and sarcopenia.' – Participant 6
	'The skin becomes atrophic, when the gut becomes aperistaltic, when there's a tremendous muscle atrophy.' – Participant 16
**Disease severity**	
Combination of activity and damage	'Severity is some combination of damage, which is irreversible, and activity, which is reversible.' – Participant 10

Disease construct and their descriptions	Exemplary quotation
Can move in either direction	“Because severity encapsulates activity, then it can potentially over time go forwards and backwards, because activity can get better or worse. And then in the background, damage can continue to be accrued.” – Participant 3
Totality of disease and its impact	‘So I think your severity score is sort of where, how bad is the scleroderma?’ – Participant 7<br><br>‘To me, it's sort of a combination of activity and damage, right? So you've got really pretty bad disease. It was very active. Like you've got a terrible polyarthritis than to that patient, I mean, the disease is severe. It could be that the damage is severe. To the patient, I mean, more damage, more severity both have an impact, right? So the patient is basically sicker and they experienced it as such, right? They experience their health quality of life as worse. They experience their function as worse. Their ability to work is less. And these can be due to either damage or activity. So severity is kind of to me a global term that encompasses everything.’ - Participant 2
Conceptually linked to distinguishing features of SSc	‘Severity to me has a concept of being perhaps more tightly linked to the fundamental pathology of scleroderma; fibrosis or vascular damage.’ – Participant 6
Clinical features of disease severity	‘I think for respiratory complications, somebody who was absolutely oxygen dependent and very severe dyspnoea and poor function due to lung fibrosis or advanced pulmonary arterial hypertension. And in a way, someone perhaps who was on home parenteral nutrition for gut failure. For example, for all those patients I can't imagine not always giving them a high severity score, whatever else was going on.’ – Participant 6<br><br>‘If they've got an active ulcer, if they've got very high skin score, if lung physiology is extremely poor.’ – Participant 9
**Overall health**	
Impact of disease	‘He's got lung fibrosis, a skin score of 15 and gets the odd ulcer. But …he’s still going out … going on family holidays …and has… joy de vivre…still enjoying life…you'd …look at a patient…and say, overall health that they're still doing okay’ – Participant 9

Disease construct and their descriptions	Exemplary quotation
Includes comorbidities	'It's not only systemic sclerosis. So, for me also other things come in if they have severe COPD, if they have like some other diseases that would be for me included in the general health.' – Participant 8
Includes side-effects	'Side effects of drugs and things come into it too. If they've got terrible gastrointestinal (symptoms) that might be from their nintedanib or from their scleroderma and they're losing weight, they've got nausea, diarrhea, they're feeling lousy, I'm going to rate that and their overall health as well because I see it as a totality.' – Participant 15
Sum of activity and damage	'Overall health is sum of activity and damage.' – Participant 12
Akin to severity	'I mean to me global health is still closer to severity in general.' – Participant 2
Disease impact	'It's not just their physical health, it's their mental health. It's the way in which they're fitting in with their social existence…with a disease like scleroderma…it's the impact… factor of their disease on their capacity to live their life.' - Participant 14

Abbreviations: COPD: chronic obstructive pulmonary disease; ILD: interstitial lung disease; NSIP: non-specific interstitial pneumonia; UIP: usual interstitial pneumonia

Supplementary Index 4: Physician perceptions of need to standardise the physician global assessment

Standardisation of PhyGA - Subthemes	Exemplary quotation
**Importance of standardisation**	
	'It's so important to our patients when we're implementing therapeutics, and we're making decisions, that we're all speaking the same language to each other and say, oh, well, this works because it helps this.' - Participant 7
	'To better standardize how people think about assessment, regardless of whether you use the term global or some other term, if we could get people to, who are assessing patients to think alike and use a construct, if you will, that can be taught and reiterated at the beginning of every clinical trial specific to the trial, then results are going to be more meaningful.' - Participant 10
**Strategies to standardise the PhyGA**	
Define construct to be measured	'I think maybe this is the most important to really have a clear definition of the question, a very defined question and then a definition of what we're actually asking for.' – Participant 8
Shared conceptual understanding of construct	'And then there has to be some understanding that the clinician has of what that conceptually means.' – Participant 3
Explicit instructions	'I could have instructions about how to weight everything.' – Participant 17
Add prompts	'You have some additional prompts to kind of guide what they should be thinking of.' – Participant 20
Define if able to review previous scores	'If you're just talking on a zero to 10 scale where you don't have the improvement, where you don't have a baseline, that's this is where the patient was last time, how are they today?...... some anchor that you have to have.' – Participant 11

**Standardisation of PhyGA - Subthemes**	**Exemplary quotation**
Training / teaching	'Offering some teaching…if we're going to attach the level of importance, we've attached on it in things like ACR-CRISS, the item should have very specific instructions…I think it is something that should require some training and, or instruction' - Participant 9
	'For clinical trials there's usually investigator meetings where for the key outcomes there's a lot of teaching and training. For example, they'll have workshops on mRSS, maybe there needs to be a workshop on physician global, right?' – Participant 20

Abbreviations: ACR-CRISS: American College of Rheumatology Composite Response Index for Systemic Sclerosis; mRSS: modified Rodnan Skin Score; PhyGA: physician global assessment
